# PanCircBase: An online resource for the exploration of circular RNAs in pancreatic islets

**DOI:** 10.3389/fcell.2022.942762

**Published:** 2022-08-19

**Authors:** Tanvi Sinha, Smruti Sambhav Mishra, Suman Singh, Amaresh Chandra Panda

**Affiliations:** ^1^ Institute of Life Sciences, Nalco Square, Bhubaneswar, Odisha, India; ^2^ Regional Center for Biotechnology, Faridabad, India

**Keywords:** pancreatic islet, divergent primer, microRNA, translation, circular RNA

## Abstract

Circular RNAs (circRNAs) are a novel class of covalently closed RNA molecules that recently emerged as a critical regulator of gene expression in development and diseases. Recent research has highlighted the importance of novel circRNAs in the biosynthesis and secretion of insulin from β-cells of pancreatic islets. However, all circRNAs expressed in pancreatic islets or β-cells are not readily available in the database. In this study, we analyzed publicly available RNA-sequencing datasets of the pancreatic islets to catalog all circRNAs expressed in pancreatic islets to construct the PanCircBase (https://www.pancircbase.net/) database that provides the following resources: 1) pancreatic islet circRNA annotation details (genomic position, host gene, exon information, splice length, sequence, other database IDs, cross-species conservation), 2) divergent primers for PCR analysis of circRNAs, 3) siRNAs for silencing of target circRNAs, 4) miRNAs associated with circRNAs, 5) possible protein-coding circRNAs and their polypeptides. In summary, this is a comprehensive online resource for exploring circRNA expression and its possible function in pancreatic β-cells.

## 1 Introduction

The last few decades have seen a significant increase in the incidences and prevalence of diabetes around the globe (Sun et al., 2022). Diabetes is often associated with a decline in insulin sensitivity or pancreatic β-cell dysfunction with decreased insulin production to maintain glucose homeostasis (Cerf, 2020). In normal conditions, pancreatic β-cells increase insulin production in response to increased glucose to maintain glucose homeostasis. However, prolonged exposure of pancreatic β-cells with high glucose or high fat leads to a decrease in insulin production by β-cell and causes diabetes (Donath et al., 2003; Cerf, 2020). Several studies established that the β-cell gene expressions are regulated at transcriptional and posttranscriptional levels. Interestingly, the genome-wide association studies identified more than a hundred type2 diabetes-associated risk loci, most of which reside in the noncoding part of the genome (Xue et al., 2018). Although most of the human genome is pervasively transcribed into RNA, only a fraction of it translates into proteins (Hangauer et al., 2013; Palazzo and Lee, 2015). The noncoding RNA family contains several types of RNAs and is mainly involved in regulating protein-coding genes. Recent studies established that long noncoding RNAs and microRNAs (miRNAs) regulate normal β-cell function and are involved in the development of diabetes (Martinez-Sanchez et al., 2016; Das et al., 2018; Wong et al., 2018). However, the role of recently discovered circular RNA (circRNA) molecules in pancreatic β-cell function has not been understood completely (Das et al., 2018; Brozzi and Regazzi, 2021).

More than 40 years ago, circRNAs were believed to be artifacts or splicing errors generating head-to-tail covalently closed RNAs without significant physiological importance (Hsu and Coca-Prados, 1979; Nigro et al., 1991; Cocquerelle et al., 1993). In the last decade, the dramatic improvements in novel computational pipelines and high throughput transcriptomic sequencing technology made it possible to realize the universal expression and function of circRNAs (Salzman et al., 2012; Jeck et al., 2013; Memczak et al., 2013). In contrast to messenger RNAs (mRNAs), circRNAs are a novel class of ubiquitously expressed closed-loop single-stranded RNA molecules without the cap and poly-A tail. Since circRNAs are highly stable, they can act as sponges for miRNAs and RNA-binding proteins (RBPs) (Panda and Xiao, 2018; Das et al., 2021). Although circRNAs are mostly categorized as noncoding RNAs, a few have been reported translating into proteins (Sinha et al., 2022). In addition, the secretion of stable circRNAs into the body fluid or exosomes makes them a promising biomarker for disease diagnosis (Lee et al., 2019). Hundreds of recent studies suggest circRNAs are associated with disease development and progression, including cancer, cardiovascular disease, rheumatoid arthritis, Alzheimer’s, and diabetes (Szabo and Salzman, 2016). Recently, a few studies highlighted the role of circRNAs in pancreatic β-cell physiology and the development of diabetes (Xu et al., 2015; Stoll et al., 2018; Das et al., 2020; Stoll et al., 2020). However, the complete landscape of circRNA expression in pancreatic islets and their involvement in diabetes is still poorly understood.

**FIGURE 1:**
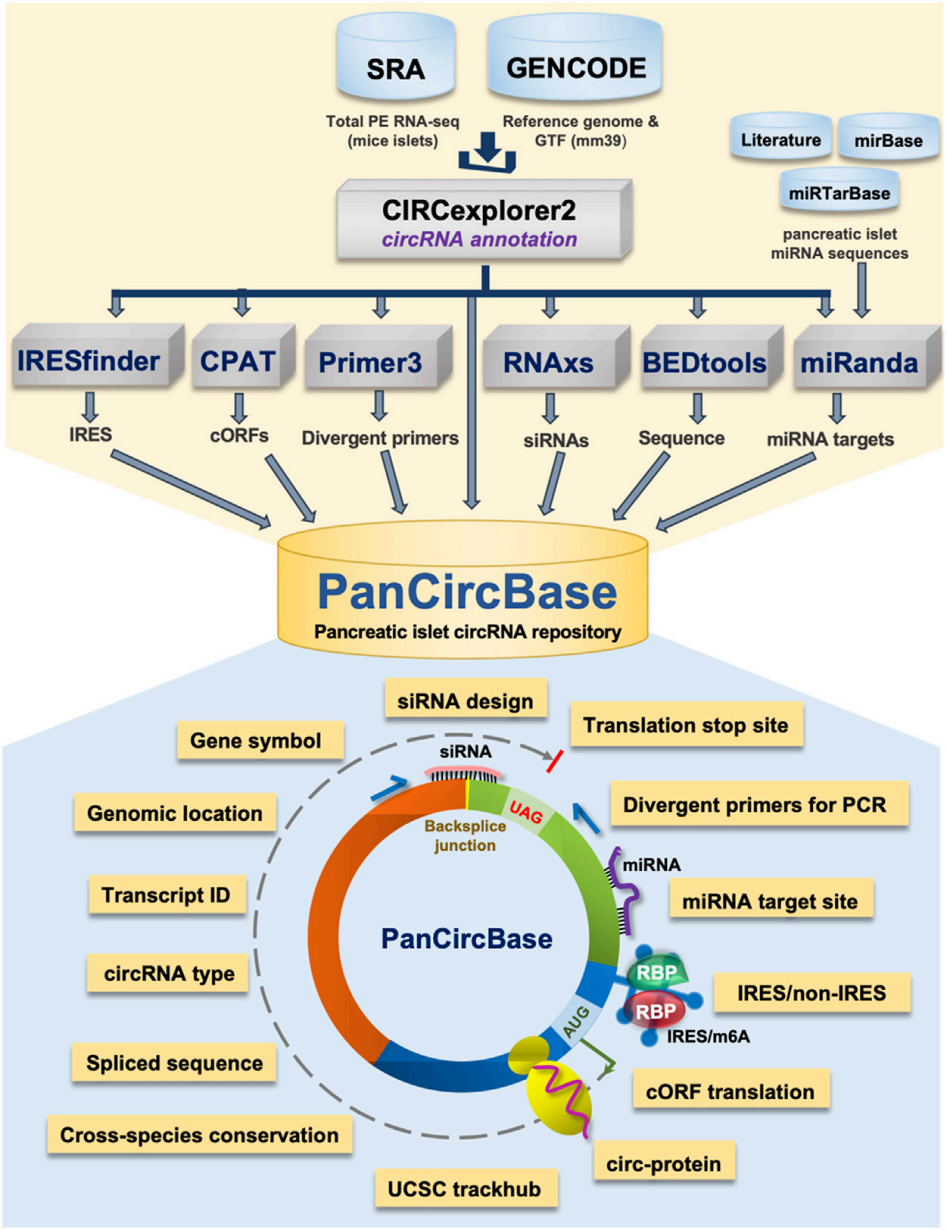
The framework of pancreatic islet circRNA database “PanCircBase”.

Given the emerging physiological importance of circRNAs and research efforts to understand them in pancreatic islet physiology, we developed the circRNA repository called PanCircBase to catalog the circRNAs expressed in pancreatic islets and provide resources for further research (Figure 1). This database presents detailed information about known and novel circRNAs expressed in pancreatic islets. Given that circRNAs regulate cellular physiology, PanCircBase provides specific details such as divergent primers, siRNAs, and miRNA targets for functional analysis of pancreatic islet circRNAs. In addition, the database provides information on the possible translation of circRNAs into proteins. In a nutshell, PanCircBase provides interactive tools to access pancreatic islet circRNA annotation details and information for functional validation of circRNAs to understand their role in ß-cell function and diabetes.

## 2 Materials and methods

### 2.1 RNA-seq data collection and circRNA analysis

We collected published total RNA-seq data of pancreatic islets of normal mice, db/db mice, or mice fed with a normal and high-fat diet (NCBI-SRA BioProject Accession No: PRJNA681104 and PRJNA358100) (Zhang et al., 2016; Motterle et al., 2017; Zhang et al., 2021). The quality of the RNA-seq reads was checked with FastQC software (v0.11.9). STAR aligner (v2.7.10a) was used to align the RNA-seq reads to the mm39 mouse genome using the ChimSegmentMin -10 parameter. The chimeric reads from the STAR aligner were used to identify the circRNAs in pancreatic islets using the CIRCexplorer2 pipeline (v2.3.8) (Zhang et al., 2016). The CIRCexplorer2 pipeline annotates genomic information of the circRNA in BED format, including chromosome number, chromosome start, chromosome end, strand, host gene symbol, transcript ID, exon information, and exon length. Furthermore, we retrieved the mature spliced circRNA sequences using BEDtools (v2.29.1). Since the pancreatic islet RNA-seq data were collected from different sources and the sequencing depth was different for different samples, we represent normalized circRNA expression levels as transcripts per million (TPM: circRNA read number/total circRNA reads in the sample × 1,000,000) along with the raw read numbers from one set of sample. The circRNA annotation data and the expression levels are provided in Supplementary Table S1. In addition, we also retrieved the IDs for pancreatic islet circRNAs in various databases such as circBase, circAtlas v2.0, CIRCpedia v2, and riboCIRC v1.0 (Glazar et al., 2014; Dong et al., 2018; Wu et al., 2020; Li et al., 2021). In addition, the conservation of PanCircBase circRNAs was analyzed by converting the circRNA chromosomal locations to human coordinates using the UCSC liftOver (Lee et al., 2022). The converted human coordinates were searched in circBase, circAtlas v2.0, and CIRCpedia v2 databases to find the human orthologs of PanCircBase circRNAs.

### 2.2 Divergent primer and siRNA design

The PCR amplicon template of circRNA backsplice junction sequences was prepared by joining 100 nt from either side of the mature sequence as described in our previous publication (Panda and Gorospe, 2018). In the case of circRNAs shorter than 200 nt, the PCR amplicon was prepared by joining the 3′ half to the 5′ half of the mature sequence. We used the Python package for the Primer3 oligo design tool (primer3-py) for designing divergent primer pairs that can amplify the backsplice junction with a PCR product ranging from 120 to 180 bp for circRNAs longer than 120 nt and PCR product range of 60–120 bp for circRNAs shorter than 120 nt (Supplementary Table S2) (Untergasser et al., 2012). Also, we applied the Tm cutoff range of 55°C–63°C and the primer length range of 18–27 nt. CircRNAs less than 60 nt in length were excluded from primer designing due to technical constraints.

The backsplice junction sequence of 26 nt spanning 13 nt on either side of the circRNA junction was used as an input in RNAxs tool for designing 19-nt siRNAs against circRNA junction sites, covering a minimum of 6 nt on either side of the backsplice junction (Supplementary Table S3) (Tafer et al., 2008).

### 2.3 Prediction of associated functional miRNAs

The miRNAs expressed in mouse pancreatic islets or β-cells were derived from previous publications, and the validated mouse functional miRNAs were retrieved from the miRTarBase release 8 (Supplementary Table S4) (Huang et al., 2022). The miRNAs with validated target genes in the miRTarBase and expressed in mouse pancreatic islets were selected for studying their association with pancreatic islet circRNAs (Huang et al., 2022). The mature miRNA sequences were downloaded from the miRBase database (Kozomara et al., 2019). The mature circRNAs sequences were used in miRanda v3.3a software to predict the functional islet miRNAs associated with target circRNAs (Betel et al., 2010).

### 2.4 Potential protein-coding circRNAs

Here, we used the CPAT v3.0.4 to analyze the protein-coding potential of pancreatic islet circRNAs (Wang et al., 2013). The CPAT scores positively correlate to the protein-coding potential of the circRNA (Supplementary Table S5). Since circRNAs are known to be translated through IRES-mediated translation initiation, we also used the IRESfinder v1.1.0 tool to predict the IRES element of pancreatic islet circRNAs (Supplementary Table S6) (Zhao et al., 2018). Moreover, the list of protein-coding mouse circRNAs was downloaded from the riboCIRC database (Li et al., 2021). The pancreatic islet circRNAs with protein-coding ability were identified from the riboCIRC database that considers the presence of potential open reading frames (ORFs), internal ribosome entry sites (IRES), m6A, mass spectrometry data of circRNA-encoded peptides as supporting evidence (Supplementary Table S7).

## 3 Results

### 3.1 Pancreatic islet circRNAs and their properties

**FIGURE 2:**
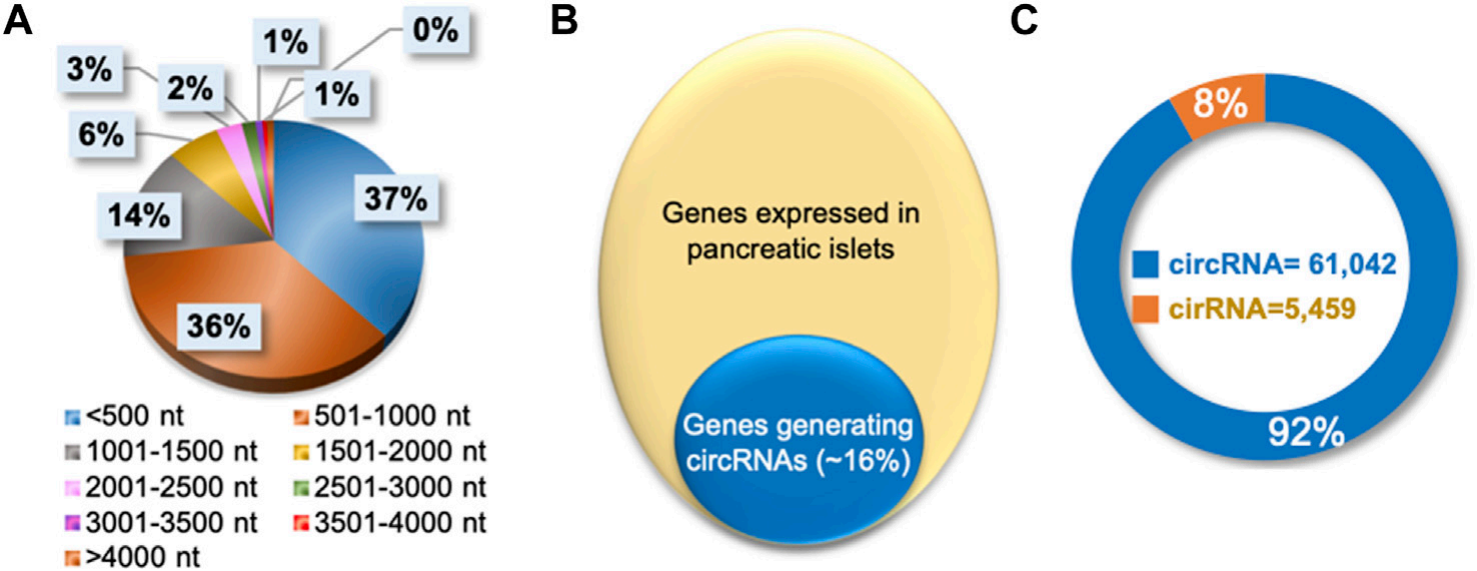
Characteristics of circRNAs expressed in pancreatic islets. **(A)**. Percentage distribution of length of circRNAs in pancreatic islets. **(B)**. Percentage of genes generating circRNAs in pancreatic islets. **(C)**. Number of circular RNAs categorized as exonic circRNA or intronic ciRNAs in pancreatic islets.

The publicly available total RNA-seq data from mice pancreatic islets were analyzed with the CIRCexplorer2 pipeline to identify circRNAs expressed in pancreatic islets (NCBI-SRA BioProject Accession No: PRJNA681104 and PRJNA358100) (Zhang et al., 2016; Motterle et al., 2017; Zhang et al., 2021). We identified 66,501 circRNAs in pancreatic islets, of which 90% were shorter than 2000 nucleotides in length (Figure 2A, Supplementary Table S1). A closer look into the identified circRNA host genes revealed that nearly 16% of genes expressed in pancreatic islets produce circular RNAs (Figure 2B). Since circRNAs are produced from intronic and exonic sequences, pancreatic islet circRNA annotation suggested that only 8% of the circular RNAs are generated solely from the intronic regions, while 92% circRNAs were generated from exons (Figure 2C). The complete list of the 66,501 circRNAs, along with their chromosomal coordinates, circRNA host gene information, splice length, and normalized expression of circRNAs in normal mice islets or high-fat-fed mice or db/db mice, are provided in Supplementary Table S1. In addition, we identified the islet circRNAs in other databases and mentioned their IDs from circBase, CIRCpedia v2, and circAtlas v2.0 in the web interface (Glazar et al., 2014; Dong et al., 2018; Wu et al., 2020). Interestingly, more than 23,000 circRNAs identified in pancreatic islets were novel and not reported in the above databases. Furthermore, we performed the conservation analysis of mouse pancreatic islet circRNAs by converting the genomic coordinates of circRNAs to the human genome coordinates using the UCSC liftOver tool and analyzed their expression in human samples in circBase, CIRCpedia v2, and circAtlas v2.0 databases (Glazar et al., 2014; Dong et al., 2018; Wu et al., 2020; Lee et al., 2022). Notably, more than half of the pancreatic islet circRNAs were expressed in human samples in the above databases suggesting their conservation in humans.

### 3.2 PanCircBase search function and detailed information on each circRNAs

**FIGURE 3:**
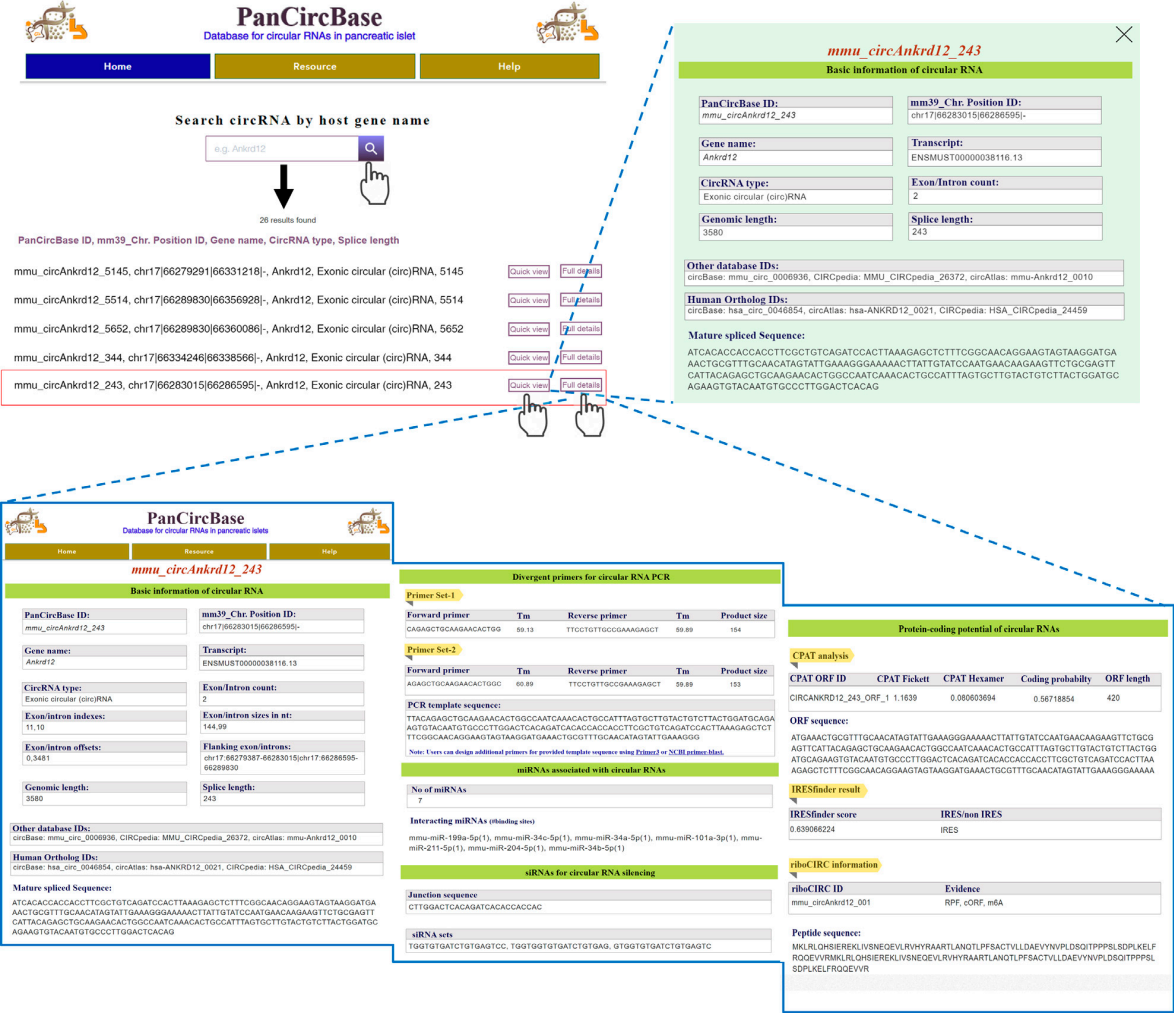
Overview of PanCircBase web interface. Users can search by the host gene symbol “Ankrd12,” The search results show all the circRNAs originating from the *Ankrd12* gene. When users go to “Quick view”, PanCircBase opens a new window with the basic information on selected circRNA, and clicking on the “Full details” option opens a new page with complete details of selected circRNA, including circRNA annotation, PCR primers, siRNAs, target miRNAs, and possible protein-coding ability of the circRNAs.

Here, the circRNAs are named based on the host gene and the mature length of the circRNAs (PanCircBase ID: gene name_circRNA length in nt). For example, the PanCircBase ID for the exonic circular (circ)RNA of 243 nt length from the *Ankrd12* gene is “*mmu_circAnkrd12_243*”. The web interface of PanCircBase is shown in Figure 3. The home page of PanCircBase contains the introduction message, related publications, and a “quick search window” for searching circRNAs by their host gene name. If a gene symbol is queried, the search results enlist all the circRNAs originating from the gene of interest in pancreatic islets. For example, the user can search for circRNAs generated from the *Ankrd12* gene upon inputting the keyword “Ankrd12” in the search box. In that case, PanCircBase search results show 26 circRNAs originating from the *Ankrd12* gene, such as *mmu_circAnkrd12_243, mmu_circAnkrd12_286, circAnkrd12_339, circAnkrd12_344* along with other circRNAs. The user can access basic annotation of each circRNA such as PanCircBase ID, chromosome position, host gene name, transcript ID, genomic length, mature spliced length, and circRNA sequence by clicking on the “Quick view” option. Alternatively, the user can click on the “Full details” button to find a detailed information page on circRNA, including PanCircBase ID, annotation details, divergent primers, interacting miRNAs, siRNAs, and protein-coding potential details of circRNA (Figure 3).

### 3.3 Resources for circRNA functional analysis

PanCircBase is a user-friendly and comprehensive database that provides useful information on 66,501 circRNAs expressed in mouse pancreatic islets. Besides the detailed circRNA information, PanCircBase also provides other information for the functional characterization of circRNAs, including divergent primers, siRNAs, associated miRNAs, and polypeptides encoded by the pancreatic islet circRNAs.

#### 3.3.1 Divergent primer for circRNA PCR

A few web tools are available for designing primers for circRNAs present in their database. For example, circInteractome can design divergent primers only for human circRNAs listed in circBase (Dudekula et al., 2016). However, no software or web server is currently available for designing divergent primers for novel circRNAs. Here, we provide two sets of specific divergent primer pairs for validation and quantification of circRNA using reverse transcription (RT) followed by PCR (RT-PCR) analysis (Supplementary Table S2). The divergent primer pairs are designed for specific PCR amplification of the target circRNA backsplice junction sequence, which can be further validated by Sanger sequencing using one of the divergent primers (Figure 3). In addition, it provides the backsplice junction PCR template sequence that can be used to design additional primer sets by using Primer3 or NCBI Primer-BLAST tool.

#### 3.3.2 siRNA for circRNA silencing

The lack of appropriate siRNA designing tools and the unavailability of circRNA junction sequences make it challenging to design siRNAs against circRNAs. We designed siRNAs targeting each pancreatic islet circRNAs using the RNAxs siRNA design tool (Tafer et al., 2008). PanCircBase provides 19-nt siRNAs targeting backsplice junction sequence spanning a minimum of six nucleotides on either side of the junction. We could successfully design 1 to 3 siRNAs for 43,483 circRNAs in the PanCircBase (Supplementary Table S3). For example, three siRNA sequences targeting the backsplice junction sequence were predicted for *mmu_circAnkrd12_243* (Figure 3). The user needs to verify the specificity of the siRNA using NCBI-BLAST. The siRNA sequences may be custom synthesized with two additional nucleotides (dTdT) as 3′ DNA overhangs.

#### 3.3.3 Predicting functional miRNA targets

Evidence suggests that circRNAs regulate gene expression by binding to miRNAs and RBPs. At present, circRNAs regulating gene expression through the circRNA-miRNA-mRNA regulatory network is the most extensively studied and accepted mechanism of circRNA-mediated gene regulation. Here, we downloaded functional miRNAs from miRTarBase and searched for pancreatic islet miRNA expression data in the literature (Poy et al., 2004; Lovis et al., 2008; Tang et al., 2009; Nesca et al., 2013; Tattikota et al., 2014; Huang et al., 2022). We retrieved the mature sequences of 478 functional miRTarBase miRNAs expressed in pancreatic islets from miRBase (Supplementary Table S4) (Kozomara et al., 2019). Systematic miRNA target site search on the pancreatic islet circRNAs using the miRanda program identified 63,505 circRNAs with at least one miRNA binding site (Betel et al., 2010). For example, *mmu_circAnkrd12_243* harbors binding sites for seven miRNAs, and each miRNA is predicted to have one binding site on *mmu_circAnkrd12_243* (Figure 3). Furthermore, we discovered that 215 circRNAs with more than 5 binding sites for a single miRNA might act as highly effective miRNA sponges (Supplementary Table S4).

#### 3.3.4 Protein-coding potential of islet circRNAs

Although circRNAs do not contain a 5′ cap and poly-A tail for conventional translation, many recent reports suggested the association of hundreds of circRNAs with polyribosomes (Sinha et al., 2022). Furthermore, ribosome footprinting assays also identified circRNAs associated with ribosomes, suggesting possible translation of circRNAs into protein products through cap-independent mechanisms (Sinha et al., 2022). Several studies have established that m6A marks or internal ribosome entry sites (IRES) on circRNA sequences promote cap-independent translation of circRNAs into functional polypeptides (Chen et al., 2016; Yang et al., 2017; Prats et al., 2020). The Coding-Potential Assessment Tool (CPAT) analysis identified 59,580 circRNAs with at least 1 ORF spanning the backsplice junction, suggesting that islet circRNAs could translate into proteins (Supplementary Table S5) (Wang et al., 2013). In addition, we could identify 40,146 pancreatic islet circRNAs with potential IRES based on IRESfinder analysis (Supplementary Table S6) (Zhao et al., 2018). For example, *mmu_circAnkrd12_243* is predicted to contain IRES by IRESfinder. Moreover, we found 475 circRNAs in PanCircBase can translate into proteins based on riboCIRC data (Supplementary Table S7) (Li et al., 2021). Interestingly, the riboCIRC database suggests that *mmu_circAnkrd12_243* (riboCIRC ID: mmu_circAnkrd12_001) can be translated into proteins based on pieces of evidence such as RPF, cORF, and m6A. Since we used a 2x circRNA sequence, the peptide sequence is truncated at the end of the circRNA sequence, while riboCIRC uses a 4x circRNA sequence, giving a longer peptide sequence.

#### 3.3.5 UCSC track for circRNAs visualization

**FIGURE 4:**
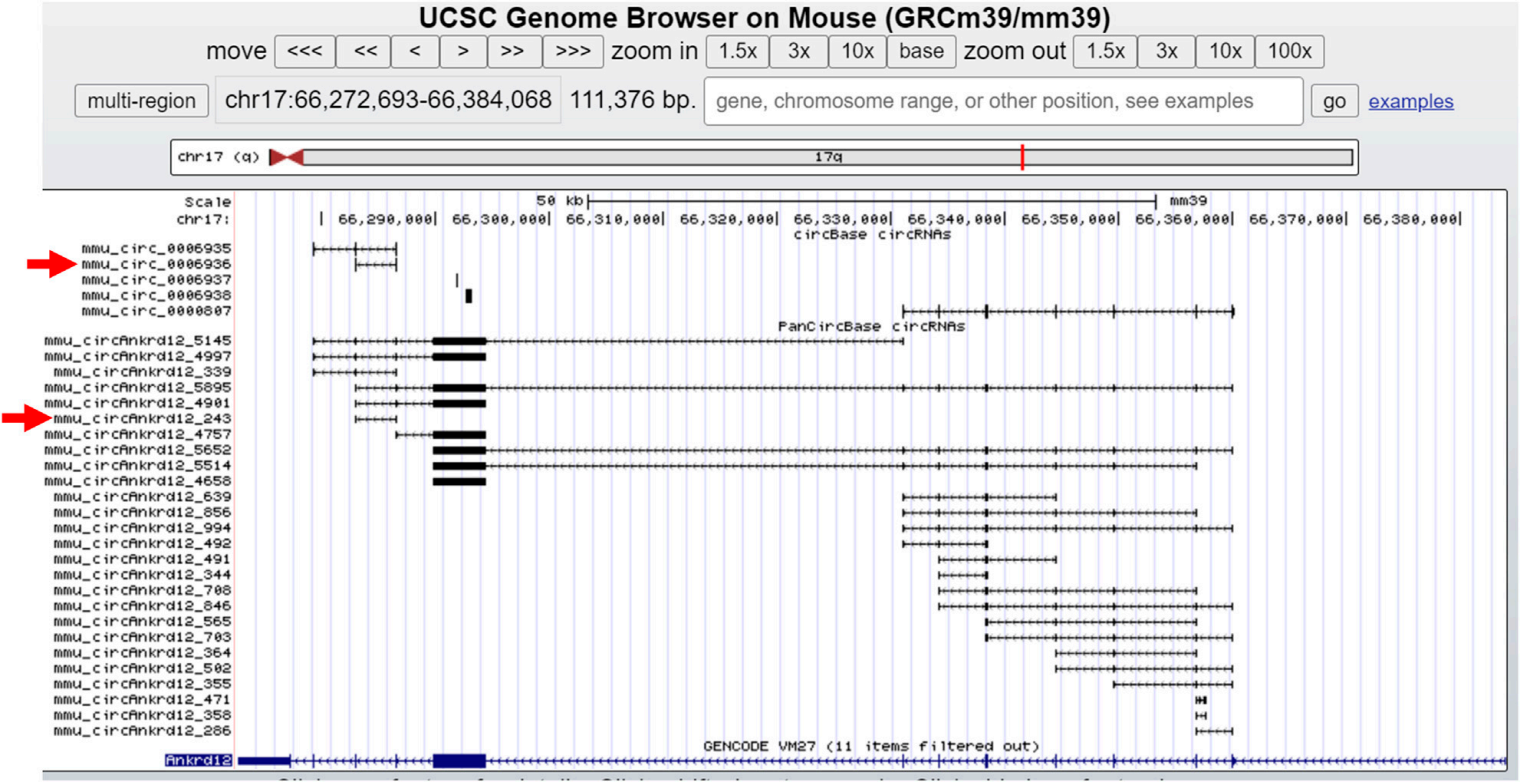
Overview of circRNAs generated from Ankrd12 in UCSC genome browser. The red arrow indicates the *mmu_circAnkrd12_243* and the corresponding circBase ID.

For direct visualization of pancreatic islet circRNAs in the UCSC genome browser, we created a link for the UCSC track of the circRNAs expressed in pancreatic islets. The user can use the link for the UCSC track provided on the resource page of PanCircBase to visualize the circRNAs generated from the gene of interest in pancreatic islets and circRNAs reported by circBase. For example, circBase reported the expression of five circRNAs from the *Ankrd12* gene, while PanCircBase identified twenty-six circRNAs generated from the Ankrd12 gene, including *mmu_circAnkrd12_243* (circBase ID: mmu_circ_0006936) (Figure 4).

## 4 Discussion

PanCircBase is a user-friendly and comprehensive database that can facilitate the study of circRNA expression and its function in pancreatic islets. PanCircBase was constructed using a collection of publicly available RNA-seq data and information from several computational tools and databases, including CIRCexplorer2, Primer3, RNAxs, miRTarBase, miRBase, miRanda, IRESfinder, CPAT, circBase, circAtlas, CIRCpedia, and riboCIRC. PanCircBase provides relevant information such as pancreatic islet circRNA annotation details, mature spliced sequences, divergent primers, circRNA-associated miRNAs, siRNAs, and potential circRNA-encoded peptides to study circRNAs. Furthermore, PanCircBase provides two sets of divergent primer pairs for PCR validation and quantification of circRNA. In addition, since circRNA silencing is one of the most relevant ways to study their function in physiological conditions, PanCircBase provides a few siRNA sequences targeting the backsplice junction sequence for specific circRNA silencing. Furthermore, open reading frames and IRES on pancreatic islet circRNAs suggest their translatability into functional proteins.

Numerous studies demonstrated the therapeutic potential of circRNAs in diseases such as cancer, neurological disorders, and cardiovascular disease using loss-of-function and gain-of-function approaches (Lee et al., 2019; Verduci et al., 2021). Recently, the dysregulation in the expression of various circRNAs has been correlated with the development of diabetes (Stoll et al., 2018; Stoll et al., 2020). Although RNA-sequencing has identified thousands of circRNAs in pancreatic islets, only a few are validated experimentally (Stoll et al., 2018; Das et al., 2020; Stoll et al., 2020). Functionally, most characterized circRNAs regulate pancreatic β-cell physiology and insulin secretion by acting as a sponge or decoy for RBPs and miRNAs (Stoll et al., 2018; Das et al., 2020; Stoll et al., 2020). Moreover, some circRNAs are secreted in the body fluid and emerge as stable biomarkers for the early detection of several diseases, including diabetes (Zaiou, 2020; Verduci et al., 2021). Together, the huge number of circRNAs reported in PanCircBase and tools to intervene and characterize circRNAs in clinical settings will help better understand the complex regulatory network of circRNAs in the development of diabetes.

Although PanCircBase provides a user-friendly platform for pancreatic islet circRNA research, it enlists the circRNAs expressed in the whole islet, including insulin-producing β-cells and other non-insulin-producing cells. Therefore, some of the circRNA reported here could also be expressed by non-insulin-producing cells. Furthermore, since the RNA-sequencing was for total RNA without depletion of linear RNAs and the circRNA sequences were predicted based on the mm39 genome annotation, the expression of circRNA splice variants with different exon/intron combinations cannot be ruled out. Given that the circRNA-associated miRNAs were predicted based on the miRanda program computationally, biochemical experiments are essential to verify functional circRNA-miRNA interaction and their downstream target gene regulation.

We will continue to maintain and update the features of PanCircBase in the foreseeable future as the new data become available. We will include circRNAs expressed in pure β-cells or in different physiological conditions as more relevant data becomes available. Furthermore, we plan to include experimentally validated functional pancreatic islet circRNAs and their regulatory network in PanCircBase. We also plan to integrate pancreatic islet or β-cell circRNAs from other species such as humans and will include the circRNA-miRNA/RBP regulatory networks. Together, PanCircBase is a valuable resource to accelerate understanding of the physiological relevance of pancreatic islet circRNAs in diabetes.

## Data Availability

The datasets presented in this study can be found in online repositories. The names of the repository/repositories and accession number(s) can be found in the article/Supplementary Material.

## References

[B1] Betel D. Koppal A. Agius P. Sander C. Leslie C. (2010). Comprehensive modeling of microRNA targets predicts functional non-conserved and non-canonical sites. Genome Biol. 11 (8), R90. 10.1186/gb-2010-11-8-r90 20799968 PMC2945792

[B2] Brozzi F. Regazzi R. (2021). Circular RNAs as novel regulators of beta-cell functions under physiological and pathological conditions. Int. J. Mol. Sci. 22 (4), 1503. 10.3390/ijms22041503 33546109 PMC7913224

[B3] Cerf M. E. (2020). Beta cell physiological dynamics and dysfunctional transitions in response to islet inflammation in obesity and diabetes. Metabolites 10 (11), E452. 10.3390/metabo10110452 33182622 PMC7697558

[B4] Chen X. Han P. Zhou T. Guo X. Song X. Li Y. (2016). circRNADb: A comprehensive database for human circular RNAs with protein-coding annotations. Sci. Rep. 6, 34985. 10.1038/srep34985 27725737 PMC5057092

[B5] Cocquerelle C. Mascrez B. Hetuin D. Bailleul B. (1993). Mis-splicing yields circular RNA molecules. FASEB J. 7 (1), 155–160. 10.1096/fasebj.7.1.7678559 7678559

[B6] Das A. Sinha T. Shyamal S. Panda A. C. (2021). Emerging role of circular RNA-protein interactions. Noncoding. RNA 7 (3), 48. 10.3390/ncrna7030048 34449657 PMC8395946

[B7] Das D. Das A. Panda A. C. (2018). Emerging role of long noncoding RNAs and circular RNAs in pancreatic β cells. Noncoding. RNA Investig. 2, 69. 10.21037/ncri.2018.11.02

[B8] Das D. Das A. Sahu M. Mishra S. S. Khan S. Bejugam P. R. (2020). Identification and characterization of circular intronic RNAs derived from insulin gene. Int. J. Mol. Sci. 21 (12), E4302. 10.3390/ijms21124302 32560282 PMC7352490

[B9] Donath M. Y. Storling J. Maedler K. Mandrup-Poulsen T. (2003). Inflammatory mediators and islet beta-cell failure: A link between type 1 and type 2 diabetes. J. Mol. Med. 81 (8), 455–470. 10.1007/s00109-003-0450-y 12879149

[B10] Dong R. Ma X. K. Li G. W. Yang L. (2018). CIRCpedia v2: An updated database for comprehensive circular RNA annotation and expression comparison. Genomics Proteomics Bioinforma. 16 (4), 226–233. 10.1016/j.gpb.2018.08.001 PMC6203687 30172046

[B11] Dudekula D. B. Panda A. C. Grammatikakis I. De S. Abdelmohsen K. Gorospe M. (2016). CircInteractome: A web tool for exploring circular RNAs and their interacting proteins and microRNAs. RNA Biol. 13 (1), 34–42. 10.1080/15476286.2015.1128065 26669964 PMC4829301

[B12] Glazar P. Papavasileiou P. Rajewsky N. (2014). circBase: a database for circular RNAs. RNA 20 (11), 1666–1670. 10.1261/rna.043687.113 25234927 PMC4201819

[B13] Hangauer M. J. Vaughn I. W. McManus M. T. (2013). Pervasive transcription of the human genome produces thousands of previously unidentified long intergenic noncoding RNAs. PLoS Genet. 9 (6), e1003569. 10.1371/journal.pgen.1003569 23818866 PMC3688513

[B14] Hsu M. T. Coca-Prados M. (1979). Electron microscopic evidence for the circular form of RNA in the cytoplasm of eukaryotic cells. Nature 280 (5720), 339–340. 10.1038/280339a0 460409

[B15] Huang H. Y. Lin Y. C. Cui S. Huang Y. Tang Y. Xu J. (2022). miRTarBase update 2022: an informative resource for experimentally validated miRNA-target interactions. Nucleic Acids Res. 50 (D1), D222–D230. 10.1093/nar/gkab1079 34850920 PMC8728135

[B16] Jeck W. R. Sorrentino J. A. Wang K. Slevin M. K. Burd C. E. Liu J. (2013). Circular RNAs are abundant, conserved, and associated with ALU repeats. RNA 19 (2), 141–157. 10.1261/rna.035667.112 23249747 PMC3543092

[B17] Kozomara A. Birgaoanu M. Griffiths-Jones S. (2019). miRBase: from microRNA sequences to function. Nucleic Acids Res. 47 (D1), D155–D162. 10.1093/nar/gky1141 30423142 PMC6323917

[B18] Lee B. T. Barber G. P. Benet-Pages A. Casper J. Clawson H. Diekhans M. (2022). The UCSC genome browser database: 2022 update. Nucleic Acids Res. 50 (D1), D1115–D1122. 10.1093/nar/gkab959 34718705 PMC8728131

[B19] Lee E. C. S. Elhassan S. A. M. Lim G. P. L. Kok W. H. Tan S. W. Leong E. N. (2019). The roles of circular RNAs in human development and diseases. Biomed. Pharmacother. 111, 198–208. 10.1016/j.biopha.2018.12.052 30583227

[B20] Li H. Xie M. Wang Y. Yang L. Xie Z. Wang H. (2021). riboCIRC: a comprehensive database of translatable circRNAs. Genome Biol. 22 (1), 79. 10.1186/s13059-021-02300-7 33685493 PMC7938571

[B21] Lovis P. Roggli E. Laybutt D. R. Gattesco S. Yang J. Y. Widmann C. (2008). Alterations in microRNA expression contribute to fatty acid-induced pancreatic beta-cell dysfunction. Diabetes 57 (10), 2728–2736. 10.2337/db07-1252 18633110 PMC2551683

[B22] Martinez-Sanchez A. Rutter G. A. Latreille M. (2016). MiRNAs in beta-cell development, identity, and disease. Front. Genet. 7, 226. 10.3389/fgene.2016.00226 28123396 PMC5225124

[B23] Memczak S. Jens M. Elefsinioti A. Torti F. Krueger J. Rybak A. (2013). Circular RNAs are a large class of animal RNAs with regulatory potency. Nature 495 (7441), 333–338. 10.1038/nature11928 23446348

[B24] Motterle A. Gattesco S. Peyot M. L. Esguerra J. L. S. Gomez-Ruiz A. Laybutt D. R. (2017). Identification of islet-enriched long noncoding RNAs contributing to beta-cell failure in type 2 diabetes. Mol. Metab. 6 (11), 1407–1418. 10.1016/j.molmet.2017.08.005 29107288 PMC5681241

[B25] Nesca V. Guay C. Jacovetti C. Menoud V. Peyot M. L. Laybutt D. R. (2013). Identification of particular groups of microRNAs that positively or negatively impact on beta cell function in obese models of type 2 diabetes. Diabetologia 56 (10), 2203–2212. 10.1007/s00125-013-2993-y 23842730

[B26] Nigro J. M. Cho K. R. Fearon E. R. Kern S. E. Ruppert J. M. Oliner J. D. (1991). Scrambled exons. Cell 64 (3), 607–613. 10.1016/0092-8674(91)90244-s 1991322

[B27] Palazzo A. F. Lee E. S. (2015). Noncoding RNA: What is functional and what is junk? Front. Genet. 6, 2. 10.3389/fgene.2015.00002 25674102 PMC4306305

[B28] Panda A. C. (2018). “Circular RNAs act as miRNA sponges,” in Circular RNAs: Biogenesis and functions. Editor Xiao J. (Singapore: Springer Singapore), 67–79.

[B29] Panda A. C. Gorospe M. (2018). Detection and analysis of circular RNAs by RT-PCR. Bio. Protoc. 8 (6), e2775. 10.21769/BioProtoc.2775 PMC5891140 29644261

[B30] Poy M. N. Eliasson L. Krutzfeldt J. Kuwajima S. Ma X. Macdonald P. E. (2004). A pancreatic islet-specific microRNA regulates insulin secretion. Nature 432 (7014), 226–230. 10.1038/nature03076 15538371

[B31] Prats A. C. David F. Diallo L. H. Roussel E. Tatin F. Garmy-Susini B. (2020). Circular RNA, the key for translation. Int. J. Mol. Sci. 21 (22), E8591. 10.3390/ijms21228591 33202605 PMC7697609

[B32] Salzman J. Gawad C. Wang P. L. Lacayo N. Brown P. O. (2012). Circular RNAs are the predominant transcript isoform from hundreds of human genes in diverse cell types. PLoS One 7 (2), e30733. 10.1371/journal.pone.0030733 22319583 PMC3270023

[B33] Sinha T. Panigrahi C. Das D. Chandra Panda A. (2022). Circular RNA translation, a path to hidden proteome. Wiley Interdiscip. Rev. RNA 13 (1), e1685. 10.1002/wrna.1685 34342387 PMC7613019

[B34] Stoll L. Rodriguez-Trejo A. Guay C. Brozzi F. Bayazit M. B. Gattesco S. (2020). A circular RNA generated from an intron of the insulin gene controls insulin secretion. Nat. Commun. 11 (1), 5611. 10.1038/s41467-020-19381-w 33154349 PMC7644714

[B35] Stoll L. Sobel J. Rodriguez-Trejo A. Guay C. Lee K. Veno M. T. (2018). Circular RNAs as novel regulators of beta-cell functions in normal and disease conditions. Mol. Metab. 9, 69–83. 10.1016/j.molmet.2018.01.010 29396373 PMC5870096

[B36] Sun H. Saeedi P. Karuranga S. Pinkepank M. Ogurtsova K. Duncan B. B. (2022). IDF Diabetes Atlas: Global, regional and country-level diabetes prevalence estimates for 2021 and projections for 2045. Diabetes Res. Clin. Pract. 183, 109119. 10.1016/j.diabres.2021.109119 34879977 PMC11057359

[B37] Szabo L. Salzman J. (2016). Detecting circular RNAs: Bioinformatic and experimental challenges. Nat. Rev. Genet. 17 (11), 679–692. 10.1038/nrg.2016.114 27739534 PMC5565156

[B38] Tafer H. Ameres S. L. Obernosterer G. Gebeshuber C. A. Schroeder R. Martinez J. (2008). The impact of target site accessibility on the design of effective siRNAs. Nat. Biotechnol. 26 (5), 578–583. 10.1038/nbt1404 18438400

[B39] Tang X. Muniappan L. Tang G. Ozcan S. (2009). Identification of glucose-regulated miRNAs from pancreatic {beta} cells reveals a role for miR-30d in insulin transcription. RNA 15 (2), 287–293. 10.1261/rna.1211209 19096044 PMC2648717

[B40] Tattikota S. G. Rathjen T. McAnulty S. J. Wessels H. H. Akerman I. van de Bunt M. (2014). Argonaute2 mediates compensatory expansion of the pancreatic beta cell. Cell Metab. 19 (1), 122–134. 10.1016/j.cmet.2013.11.015 24361012 PMC3945818

[B41] Untergasser A. Cutcutache I. Koressaar T. Ye J. Faircloth B. C. Remm M. (2012). Primer3--new capabilities and interfaces. Nucleic Acids Res. 40 (15), e115. 10.1093/nar/gks596 22730293 PMC3424584

[B42] Verduci L. Tarcitano E. Strano S. Yarden Y. Blandino G. (2021). CircRNAs: Role in human diseases and potential use as biomarkers. Cell Death Dis. 12 (5), 468. 10.1038/s41419-021-03743-3 33976116 PMC8113373

[B43] Wang L. Park H. J. Dasari S. Wang S. Kocher J. P. Li W. (2013). CPAT: Coding-Potential Assessment Tool using an alignment-free logistic regression model. Nucleic Acids Res. 41 (6), e74. 10.1093/nar/gkt006 23335781 PMC3616698

[B44] Wong W. K. M. Sorensen A. E. Joglekar M. V. Hardikar A. A. Dalgaard L. T. (2018). Noncoding RNA in pancreas and beta-cell development. Noncoding. RNA 4 (4), E41. 10.3390/ncrna4040041 30551650 PMC6315983

[B45] Wu W. Ji P. Zhao F. (2020). CircAtlas: An integrated resource of one million highly accurate circular RNAs from 1070 vertebrate transcriptomes. Genome Biol. 21 (1), 101. 10.1186/s13059-020-02018-y 32345360 PMC7187532

[B46] Xu H. Guo S. Li W. Yu P. (2015). The circular RNA Cdr1as, via miR-7 and its targets, regulates insulin transcription and secretion in islet cells. Sci. Rep. 5, 12453. 10.1038/srep12453 26211738 PMC4515639

[B47] Xue A. Wu Y. Zhu Z. Zhang F. Kemper K. E. Zheng Z. (2018). Genome-wide association analyses identify 143 risk variants and putative regulatory mechanisms for type 2 diabetes. Nat. Commun. 9 (1), 2941. 10.1038/s41467-018-04951-w 30054458 PMC6063971

[B48] Yang Y. Fan X. Mao M. Song X. Wu P. Zhang Y. (2017). Extensive translation of circular RNAs driven by N(6)-methyladenosine. Cell Res. 27 (5), 626–641. 10.1038/cr.2017.31 28281539 PMC5520850

[B49] Zaiou M. (2020). circRNAs signature as potential diagnostic and prognostic biomarker for diabetes mellitus and related cardiovascular complications. Cells 9 (3), E659. 10.3390/cells9030659 32182790 PMC7140626

[B50] Zhang F. Yang Y. Chen X. Liu Y. Hu Q. Huang B. (2021). The long non-coding RNA βFaar regulates islet β-cell function and survival during obesity in mice. Nat. Commun. 12 (1), 3997. 10.1038/s41467-021-24302-6 34183666 PMC8238983

[B51] Zhang X. O. Dong R. Zhang Y. Zhang J. L. Luo Z. Zhang J. (2016). Diverse alternative back-splicing and alternative splicing landscape of circular RNAs. Genome Res. 26 (9), 1277–1287. 10.1101/gr.202895.115 27365365 PMC5052039

[B52] Zhao J. Wu J. Xu T. Yang Q. He J. Song X. (2018). IRESfinder: Identifying RNA internal ribosome entry site in eukaryotic cell using framed k-mer features. J. Genet. Genomics 45 (7), 403–406. 10.1016/j.jgg.2018.07.006 30054216

